# Platelet Aggregation Alterations in Patients with Severe Viral Infection Treated at the Intensive Care Unit: Implications for Mortality Risk

**DOI:** 10.3390/pathogens13090778

**Published:** 2024-09-10

**Authors:** Wojciech Bakowski, Jakub Smiechowicz, Barbara Dragan, Waldemar Goździk, Barbara Adamik

**Affiliations:** Clinical Department of Anesthesiology and Intensive Therapy, Wroclaw Medical University, Borowska 213, 50-556 Wroclaw, Poland; wojciech.bakowski.umed@gmail.com (W.B.); jakub.smiechowicz@umw.edu.pl (J.S.); barbara.dragan@umw.edu.pl (B.D.); waldemar.gozdzik@umw.edu.pl (W.G.)

**Keywords:** platelet aggregation, platelet desensitization, COVID-19, intensive care, viral sepsis, ARDS, ECMO

## Abstract

Severe viral infections often result in abnormal platelet function, affecting various stages of hemostasis. Activated platelets are often considered prothrombotic and more susceptible to further stimulation. However, emerging evidence suggests that initial hyperactivation is followed by platelet exhaustion and hypo-responsiveness, affecting platelet degranulation, activation, and aggregation. We examined early alterations in platelet aggregation among patients (N = 28) with acute respiratory distress syndrome and SARS-CoV-2 infection who were receiving mechanical ventilation and venovenous extracorporeal membrane oxygenation support. Blood samples were stimulated with four different activators: arachidonic acid, adenosine diphosphate, thrombin receptor-activating protein 6, and ristocetin. Our observations revealed that platelet aggregation was reduced in most patients upon admission (ranging from 61 to 89%, depending on the agonist used), and this trend intensified during the 5-day observation period. Concurrently, other coagulation parameters remained within normal ranges, except for elevated d-dimer and fibrinogen levels. Importantly, we found a significant association between platelet aggregation and patient mortality. Impaired platelet aggregation was more severe in patients who ultimately died, and reduced aggregation was associated with a significantly lower probability of survival, as confirmed by Kaplan–Meier analysis (*p* = 0.028). These findings underscore the potential of aggregometry as an early detection tool for identifying patients at higher risk of mortality within this specific cohort.

## 1. Introduction

Platelets play a pivotal role in both hemostasis and the immune response to infection. Alterations in platelet count, morphology, and function can significantly impact prognosis. During severe infections, inflammatory mediators activate coagulation pathways, leading to platelet activation. Additionally, pathogens can directly activate platelets by releasing toxins or indirectly through complex formation with plasma proteins, activating platelet receptors [1]. Activated platelets engage in aggregation with leukocytes and vascular endothelial cells, aiding in pathogen elimination through processes like phagocytosis and extracellular neutrophil traps.

Notably, SARS-CoV-2 infections often result in abnormal platelet function, affecting various stages of hemostasis [2]. The virus primarily binds to angiotensin-converting enzyme 2 (ACE2), which facilitates cell entry via the viral spike protein [3]. Transmembrane protease subtype 2 (TMPRSS2) assists in this process, and both ACE2 and TMPRSS2 are expressed in cells, including platelets [4]. The underlying mechanism of platelet dysfunction during viral infections is multifaceted. SARS-CoV-2 binding and internalization trigger platelet degranulation, secretion, integrin receptor activation, and aggregation. This prothrombotic and proinflammatory state may elevate the risk of thrombotic complications [5,6]. However, platelet activation also leads to increased consumption and degradation, potentially contributing to thrombocytopenia. Emerging evidence suggests that initial hyperactivation is followed by platelet exhaustion and hypo-responsiveness, affecting degranulation, GPIIb/IIIa activation, and aggregation [6,7,8].

Extracorporeal membrane oxygenation (ECMO) is a life-saving procedure employed in patients experiencing acute respiratory or circulatory failure, particularly those with acute respiratory distress syndrome (ARDS) due to SARS-CoV-2 infection [9]. During ECMO therapy, non-physiological shear stress arises from blood contact with artificial surfaces or abnormal blood flow, potentially disrupting hemostasis [10]. These shear forces trigger interactions between the platelet receptor GPIbα and immobilized von Willebrand factor (VWF) on the extracellular matrix. Consequently, the integrin GPIIb/IIIa undergoes ligand-binding activation, leading to platelet adhesion, aggregation, and clot formation [11]. Additionally, non-physiological shear stress modulates direct interactions between VWF and GPIIb/IIIa, promoting platelet aggregation independent of the VWF–GPIbα–GPIIb/IIIa pathway [12,13]. Recent studies have revealed that excessive shear stress can also induce shedding of platelet adhesion receptors, specifically GPIbα and GPVI. Loss of these critical receptors on the platelet surface reduces their capacity for adhesion and aggregation, potentially contributing to coagulation abnormalities [14,15].

In our study, we examined early alterations in platelet aggregation among patients with ARDS and SARS-CoV-2 infection who were receiving venovenous extracorporeal membrane oxygenation (VV-ECMO) support. Our hypothesis was that abnormal platelet aggregation in this cohort might influence patient survival in the intensive care unit.

## 2. Materials and Methods

### 2.1. Patients

The data for this single-center, retrospective, observational study were extracted from hospital records of patients treated at the intensive care unit (ICU) of Wroclaw University Hospital between October 2020 and March 2022 (spanning 18 months). The study protocol received approval from the Bioethics Committee of Wroclaw Medical University (Approval No. KB–996/2021), and the research adhered to the principles outlined in the 1975 Declaration of Helsinki, as amended in 2008. Informed consent was waived by the Bioethics Committee due to the retrospective nature of this study. The inclusion criteria encompassed patients with confirmed SARS-CoV-2 infection based on positive results from real-time reverse-transcriptase polymerase chain reaction tests of nasal or pharyngeal swab samples before ICU admission. Additionally, patients needed a diagnosis of acute respiratory distress syndrome (ARDS) according to the Berlin definition criteria [16]. ECMO therapy initiation on the day of ICU admission and availability of aggregometry test results were also prerequisites for inclusion. Exclusion criteria comprised an age below 18 years, the absence of platelet aggregation test results, or a history of congenital, acquired, or other known coagulopathies.

### 2.2. Data Collection

Clinical and laboratory parameters were routinely assessed on days 1, 3, and 5 of ICU stay. Organ failure monitoring utilized the Sequential Organ Failure Assessment (SOFA) score, evaluating cardiovascular (mean arterial pressure and vasopressor dosage), respiratory (PaO_2_/FiO_2_ ratio), hepatic (bilirubin concentration), renal (creatinine concentration), neurological (Glasgow coma scale), and coagulation (platelet count) systems. The Acute Physiology and Chronic Health Evaluation (APACHE II) scale was used on admission to the ICU to determine the patient’s clinical status. To calculate the APACHE II score for each patient, the laboratory parameters, such as blood pH, serum sodium, potassium, creatinine, hematocrit, white blood cells, oxygen partial pressure, inhaled oxygen fraction, and values of clinical parameters, such as the Glasgow coma scale, heart rate, respiratory rate, body temperature, and mean arterial pressure, were used. In addition, the following data were collected from each patient on days 1, 3, and 5 of ICU treatment: platelet count, d-dimer, fibrinogen, antithrombin III (AT III), and thromboelastometry results. Thromboelastometry was performed using the ROTEM delta system (Pentapharm, Munich, Germany) within 30 min of blood sample collection. All reagents used for thromboelastometry tests were commercially available and CE-IVD marked. The extrinsically activated test (EXTEM) was conducted by recalcifying citrated blood (300 µL) with 20 µL of 0.2 mol/L CaCl2 and stimulating it with tissue factor. The intrinsically activated test (INTEM) was conducted by recalcifying blood (300 µL) with CaCl2 and stimulating it with thromboplastin. The thromboelastometric parameters analyzed included clotting time (CT), clot formation time (CFT), and maximum clot firmness (MCF). CT is defined as the time from the initiation of clot formation until a 2 mm amplitude is achieved. CFT is the time from a 2 mm to 20 mm amplitude of the clotting signal. MCF is the parameter indicating the strength and firmness of the clot that is reached before the clot is dissolved by fibrinolysis. Reference ranges for the ROTEM parameters were provided by the manufacturer.

### 2.3. Platelet Aggregation Measurements

To assess platelet aggregation, we conducted platelet receptor function analysis using the agonists arachidonic acid (ASPI), adenosine diphosphate (ADP), thrombin receptor-activating protein (TRAP), and ristocetin (RISTO) upon admission to the ICU and on days 3 and 5. Blood samples for aggregometry were collected from the arterial line using a vacutainer system and 3 mL tubes containing hirudin as an anticoagulant (Roche Diagnostics GmbH, Mannheim, Germany). Whole blood impedance aggregometry was performed using the Multiplate analyzer (Roche Diagnostics GmbH, Mannheim, Germany). The Multiplate analyzer features four separate chambers, each designated for a different platelet activator, allowing simultaneous use, with each measurement taking six minutes to complete. Platelet aggregation was assessed by measuring changes in electrode conductivity caused by the continuous formation of platelet aggregates over the six-minute period. The results are expressed as the mean area under the curve (AUC) from two electrodes and are presented in units (AU/min). The reference ranges provided by the manufacturer are as follows: ADP test: 534–1220 AU/min, TRAP test: 941–1536 AU/min, RISTO test: 896–2013 AU/min, and ASPI test: 745–1361 AU/min. According to the manufacturer’s protocol, all measurements were conducted within two hours post-blood collection, immediately following a complete blood count, as aggregometry requires a platelet count above 100 × 10^9^/L in the sample. The results of impedance aggregometry measured in this study were analyzed in relation to reference values.

For this experiment, we employed CE-IVD (European Conformity—in vitro diagnostic)-certified tests utilizing four different agonists to stimulate specific receptors on the platelets. (1.) For the ASPI test, arachidonic acid (0.5 mM) was added to the blood sample. Arachidonic acid is converted to prostaglandin H2 (PGH2) by cyclooxygenase-1 (COX1), and PGH2 is subsequently converted to thromboxane A2 (TXA2) by thromboxane synthase. TXA2 enhances platelet aggregation and stimulates platelet degranulation and activation. (2.) For the ADP test, adenosine diphosphate (6.5 μM) was added to the blood sample. ADP induces platelet shape change and degranulation, with the released granule contents further activating the platelets. Activation also causes a conformational change in the glycoprotein IIb/IIIa (GPIIb/IIIa) receptor, increasing its affinity for fibrinogen. The binding of fibrinogen to GPIIb/IIIa results in platelet aggregation. (3.) For the TRAP test, thrombin receptor-activating protein 6 (32 μmol/L) was added to activate the thrombin receptor PAR-1 on platelets. TRAP mimics the action of thrombin, a potent activator of platelet aggregation. (4.) For the RISTO test, ristocetin (0.77 mg/mL) was added. Ristocetin forms complexes with von Willebrand factor (vWF). The binding of ristocetin–vWF complexes leads to the aggregation and activation of platelets.

### 2.4. Patient Management

The management of patients included standard treatment for sepsis, extracorporeal membrane oxygenation (ECMO), and other supportive therapies, adhering to established guidelines. The primary sepsis management, including the use of vasopressors, fluid therapy, steroids, antibiotics, and continuous renal replacement therapy, was conducted in accordance with the Surviving Sepsis Campaign guidelines [17]. The decision to implement venovenous ECMO (V-V ECMO) was made by the ECMO team of the ICU Department at Wroclaw University Hospital based on the Extracorporeal Life Support Organization guidelines [18]. In most cases, ECMO was established in the ICU, except for a few patients who were cannulated by the ECMO team in another facility and transported on ECMO to the ICU at Wroclaw University Hospital. Inclusion criteria for V-V ECMO therapy were based on current guidelines and included persistent hypoxemia with PaO_2_/FiO_2_ < 150 mmHg and/or respiratory acidosis with pH < 7.25 and PaCO_2_ > 60 mmHg despite conventional ARDS therapy, protective lung ventilation, muscle relaxants, and the prone positioning [19]. ECMO cannulation was performed percutaneously. The V-V ECMO circuit included a Quadrox-i adult microporous membrane oxygenator (MAQUET Holding BV & Co, KG, Rastatt, Germany) and either a CardioHelp or continuous life support set with a Rotaflow II base unit (MAQUET Holding BV & Co, KG, Germany). Verification of proper cannula positioning was performed via a chest X-ray, ultrasound examination, and clinical assessment of the patient’s condition. The general goal of ECMO therapy was to prevent hypoxemia and hypercapnia, maintain oxygen saturation above 88%, normoxemia, normocapnia in arterial blood gas, and prevent acid–base disorders. Multimodal analgosedation was implemented and was guided by the Richmond Agitation and Sedation Scale (RASS). All patients included in this study received systemic anticoagulation with a continuous infusion of unfractionated heparin. ECMO settings depended on the patient’s clinical condition, with a sweep gas flow of 3–5 L/min used in most patients. The settings of the centrifugal pump were usually in the range of 3–5 L/min. Patients were assessed daily for possible ECMO weaning using the End of Life Options Act clinical and physiological criteria [18].

### 2.5. Statistical Analysis

All analyses were conducted using Statistica version 13.0, licensed to Wroclaw Medical University, Wroclaw, Poland. Continuous variables are presented as medians with 25th and 75th percentiles, while categorical variables are summarized as counts and fractions. The Shapiro–Wilk test indicated that the distribution of variables was not normal. Consequently, nonparametric techniques were employed for statistical analysis. Differences between groups were assessed using the Mann–Whitney U test for continuous variables. Categorical variables were analyzed using the chi-squared test. The Friedman repeated-measures ANOVA was used to analyze changes in aggregometry test results over time. Receiver operating characteristic (ROC) curve analysis was utilized to evaluate the discriminative ability of the aggregometry parameters measured in whole blood to distinguish between ICU deaths and survivors. This was performed by calculating the area under the curve (AUC), including 95% confidence intervals (CIs), to determine sensitivity and specificity. Survival analysis of time to death was performed using the Kaplan–Meier curve and a log-rank test. Statistical significance was assumed if the probability of the null hypothesis was ≤5% (*p* ≤ 0.05).

## 3. Results

### 3.1. Characterization of the Patient Cohort

From October 2020 to March 2022 (18 months), a total of 46 consecutive patients with acute respiratory distress syndrome (ARDS) treated with V-V ECMO were screened for inclusion/exclusion criteria. Among them, the 28 patients who underwent platelet aggregation testing were included in the final analysis. The median age of patients was 44 years (IQR: 35–55), and the majority were male (89%). ARDS and sepsis of viral origin, according to the Sepsis-3 definition, were diagnosed in all patients upon ICU admission [20]. None of the included patients had a history of coexisting diseases that would necessitate intensive anticoagulant treatment. Venous thromboembolism prophylaxis was prescribed to most of the COVID-19 patients as a standard of care in the ICU. The median ICU stay was 24 days, with an ICU mortality rate of 54%, and the median hospital stay was 26 days, with a hospital mortality rate of 57%. Based on ICU survival status, patients were divided into two groups: those who survived (N = 13) and those who died (N = 15). Patient baseline characteristics are presented in Table 1. ECMO treatment was initiated for each patient within 24 h of admission to the ICU. All patients had a PaO_2_/FiO_2_ ratio below 100. The median duration of ECMO was 14 days (IQR: 11–21) in patients who survived and 13 days (IQR: 7–21) in patients who died, with no significant difference between the groups (*p* = 0.746). The reason for ECMO termination was the improvement of clinical status in 13 patients and death in 15 patients. All patients who were successfully weaned from ECMO support were discharged from the ICU in stable condition. Of these, one patient later died of thromboembolism in another hospital ward. ECMO hemorrhagic complications were analyzed according to the definitions of the ELSO Registry. During the 5-day observation period, none of the patients experienced any hemorrhagic complications associated with ECMO or as a consequence of ECMO; none of the patients required packed red blood cell or whole blood transfusion or other interventions. The median Hb level was 11.7 g/dL (IQR: 10.4–12.9), 10.4 g/dL (IQR: 9.9–11.0), and 10.0 g/dL (IQR: 9.3–11.8) on days 1, 3, and 5, respectively, with no statistically significant differences between survivors and nonsurvivors.

### 3.2. Patients Who Ultimately Died Exhibited Decreased Platelet Aggregation

To assess the ability of platelets to aggregate, we analyzed the results of aggregometry tests performed on the 1st, 3rd, and 5th days of treatment in the ICU. Blood samples were stimulated with four different activators: arachidonic acid for the ASPI test, adenosine diphosphate for the ADP test, thrombin receptor-activating protein 6 for the TRAP test, and ristocetin for the RISTO test. The results were analyzed with respect to the reference range provided by the manufacturer for each test. We observed that platelet aggregation in most patients (61–89%, depending on the agonist used) was below the reference range for all tested agonists on the 1st day of ICU treatment, and these alterations persisted on the 3rd and 5th days (Table 2). However, analysis of changes in aggregometry test results over time using Friedman repeated-measures ANOVA showed no significant changes in ASPI (*p* = 0.400), ADP (*p* = 0.146), TRAP (*p* = 0.105), and RISTO (*p* = 0.643) test results.

**Figure 1:**
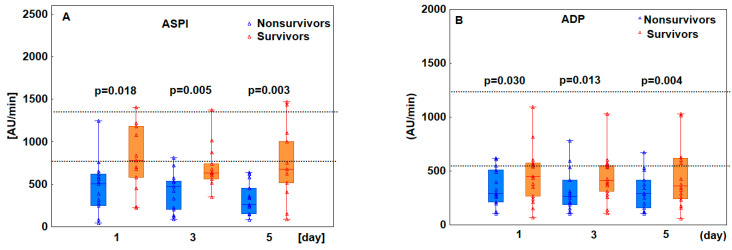

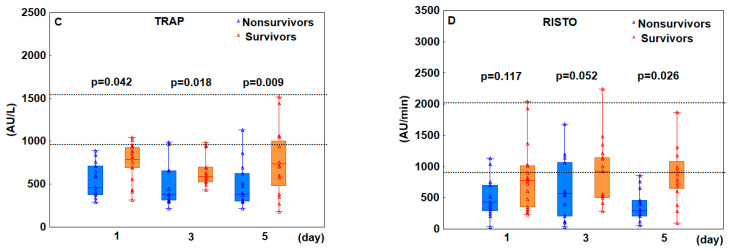
Graphs comparing platelet receptor activity between survivors and nonsurvivors on days 1, 3, and 5 of ECMO treatment. Dotted lines represent the lower and upper reference ranges for each test: ASPI 745–1361 AU/min (**A**), ADP 534–1220 AU/min (**B**), TRAP 941–1536 AU/min (**C**), RISTO 896–2013 AU/min (**D**). The *p*-values represent differences between the study groups at each time point. The box plots represent the median values (middle line) with upper and lower quartiles (box); the whiskers represent the minimum and maximum values.

We then assessed whether there was a difference in platelet receptor response between patients who survived and those who died. On day 1, median platelet receptor activation was significantly lower in nonsurvivors compared to survivors in all tests, except RISTO, indicating abnormal platelet aggregation. On days 3 and 5, platelet response to all agonists remained below normal, with significantly lower values in nonsurvivors compared to survivors (Figure 1).

### 3.3. Other Parameters of Coagulation Were Normal except for D-Dimer and Fibrinogen Levels

The median platelet count was normal in both groups throughout this study, with no significant differences between survivors and nonsurvivors. On day 1, all patients had platelet counts within the reference range. Among survivors, 8% had platelet counts below normal on day 3 and 15% on day 5, while among nonsurvivors, 20% had platelet counts below normal on day 3 and 47% on day 5. In survivors, the minimum and maximum platelet counts were 169.0–553.0, 132.0–406.0, and 106.0–302.0 × 10^9^/L, and in nonsurvivors, the counts were 137.0–418.0, 105.0–344.0, and 102.0–314.0 × 10^9^/L on days 1, 3, and 5, respectively. D-dimer and fibrinogen levels were elevated in both groups on days 1, 3, and 5, with no significant differences between groups, except for d-dimer levels, which were significantly lower in survivors than in nonsurvivors on day 3 (2.3 vs. 6.5 mg/L, *p* = 0.021) and day 5 (4.5 vs. 10.8 mg/L, *p* = 0.024). The results of standard coagulation parameters are presented in Table 3.

Additionally, EXTEM (extrinsic activation of coagulation) and INTEM (intrinsic activation of coagulation) thromboelastometric tests were analyzed to assess clot formation and strength. Clotting time (CT), clot formation time (CFT), and maximum clot firmness (MCF) were compared between survivors and nonsurvivors; median values for these parameters were similar between survivors and nonsurvivors, with no statistically significant differences on any study day (Table 4).

### 3.4. Decreased Platelet Aggregation Results Predicted Poor Prognosis

**Figure 2:**
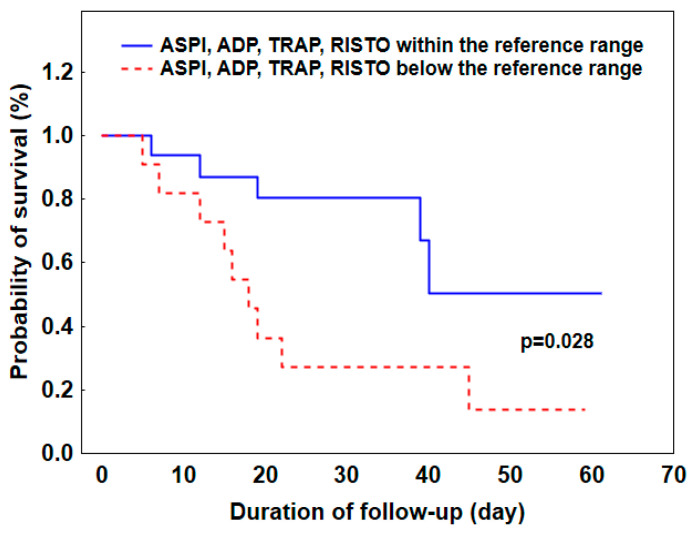
Kaplan–Meier curves stratified by the presence of platelet aggregation results recorded on day 1 in all tests (ASPI, ADP, TRAP, and RISTO) below vs. within the reference range (log-rank test).

On day 1, platelet aggregation results below the reference range for all tests (ASPI, ADP, TRAP, and RISTO) were recorded in 43% of patients. By day 3, this percentage increased to 54%, and by day 5, it reached 64%. Importantly, the presence of platelet aggregation results below the reference range for all tests on day 1 was associated with a significantly worse prognosis of survival (log-rank test *p* = 0.028, Figure 2).

**Figure 3:**
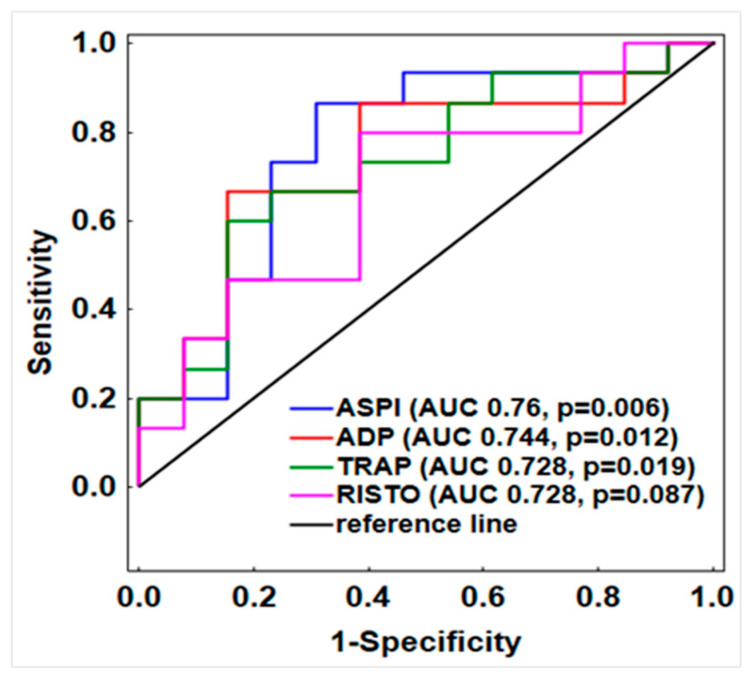
ROC curve statistics for aggregometry results measured on day 1 as predictors of ICU mortality. AUC, area under the curve.

To investigate the aggregometry test results as potential predictors of ICU mortality in the study cohort, receiver operating characteristic (ROC) curves were generated and areas under the curve (AUC) and significance levels were obtained from these curves as measures of test reliability (Figure 3). Based on ROC curve analysis, 1st day ASPI, ADP, and TRAP test results showed good predictive ability for ICU mortality. The best discriminatory cut-off value calculated with Youden’s Statistic for the ASPI test was 654 AU/min (sensitivity 0.867, specificity 0.692), ADP was 396 AU/min (sensitivity 0.667, specificity 0.846), and TRAP was 586 AU/min (sensitivity 0.600, specificity 0.846).

## 4. Discussion

In this study, we evaluated early changes in platelet aggregation among patients with acute lung injury following viral infection. This study encompassed patients diagnosed with ARDS induced by SARS-CoV-2 infection who, upon ICU admission, necessitated mechanical ventilation and ECMO to support lung function. Our observations revealed that platelet aggregation was reduced in most patients upon admission, and these alterations persisted during the 5-day observation period. Concurrently, other coagulation parameters remained within normal ranges, with the exception of increased d-dimer and fibrinogen levels. Importantly, we found a significant association between platelet aggregation and patient mortality. Impaired platelet aggregation was more severe in patients who ultimately died, and reduced aggregation was associated with a significantly lower probability of survival, as confirmed by Kaplan–Meier analysis. These findings underscore the potential of aggregometry as an early detection tool for identifying patients at higher risk of mortality within this specific cohort.

Platelets are crucial for hemostasis and endothelial integrity and play a significant role in the innate immune response to viral infections [21]. They express various receptors, including toll-like receptors, which detect viral pathogen-associated molecular patterns. The stimulation of these receptors can lead to platelet activation, granular secretion, and aggregation [22,23]. Studies on platelet aggregation in patients with COVID-19 have yielded conflicting results, often due to methodological differences such as varying agonist concentrations, measurement methods, and the use of whole blood versus platelet-rich plasma samples. In our study, platelet aggregation was measured using standardized whole blood impedance aggregometry tests (CE-IVD certified) and a commercially available analyzer routinely used in the ICU, other hospital wards, and operating theatres. The impedance aggregometry method analyzes whole blood samples, thereby eliminating the need for platelet-rich plasma. This approach is advantageous as platelets are highly sensitive to mechanical or biological stimulation and can be easily activated during the preparation of platelet-rich plasma, such as through centrifugation. In our study, in the cohort of critically ill COVID-19 patients with ARDS, decreased platelet aggregation in response to various agonists such as arachidonic acid, adenosine diphosphate, thrombin receptor-activating protein 6, and ristocetin was observed in most patients over a 5-day observation period. At the same time, d-dimer and fibrinogen levels increased, indicating earlier activation of coagulation. D-dimer, a specific degradation product of stabilized fibrin, is elevated in conditions reflecting thrombin generation and degradation. Previous studies indicate that elevated d-dimer levels may be associated with coagulopathy, worse clinical conditions, and higher mortality rates [24,25]. Consistent with previous studies, this study found significantly higher d-dimer levels in patients who died compared to those who survived. Interestingly, similar findings were noted in patients during the early stages of SARS-CoV-2 infection. In a study by Riedel et al., who assessed platelet reactivity in patients with mild to moderate disease severity (non-ICU patients) at early stages of SARS-CoV-2 infection, impaired platelet aggregation was found in response to arachidonic acid, adenosine diphosphate, and thrombin receptor-activating protein 6 [26]. Moreover, decreased platelet responsiveness was associated with impaired activation of GPIIb/IIIa, a transmembrane platelet receptor that, when activated, causes platelet aggregation by binding to fibrinogen and von Willebrand factor. Similarly, Bertolin et al. showed lower platelet reactivity in response to arachidonic acid and thrombin receptor-activating protein 6 and, to a lesser extent, to adenosine diphosphate, in patients with mild COVID-19 [27]. At the same time, levels of d-dimer, fibrinogen, and plasminogen activator inhibitor 1 were increased, indicating the activation of coagulation and the inhibition of fibrinolysis. In contrast to these findings, Heintz et al. evaluated critically ill ICU patients with COVID-19 and ARDS, discovering reduced platelet aggregation only in response to adenosine diphosphate, while aggregation remained normal after stimulation with arachidonic acid or thrombin receptor-activating protein 6 [28]. At the same time, the levels of d-dimer and von Willebrand antigen increased which, considering previous findings, may indicate endothelial damage [29,30]. The discrepancies between Riedel’s findings and our own may be attributed to the timing of aggregometric tests. Riedel’s study conducted these tests approximately 7 days post-ICU admission, whereas our study monitored patients from the day of ICU admission through the 5th day. The extended ICU treatment period in Riedel’s study could have enhanced the patients’ clinical conditions, thereby affecting the aggregometry outcomes. Changes in platelet aggregation are not exclusive to viral infections; critically ill ICU patients with bacterial infections also exhibit abnormal platelet aggregation. Several previous studies have demonstrated significantly reduced platelet aggregation in response to various agonists in patients with bacterial sepsis, correlating with a worse prognosis [31]. Davies et al. observed a significant reduction in platelet aggregation in response to arachidonic acid or adenosine diphosphate in sepsis patients; moreover, a reduction in aggregation was significantly associated with increased sepsis severity and higher ICU mortality rates [32]. Similarly, Laursen et al. found that platelet aggregation, when stimulated by adenosine diphosphate or thrombin receptor-activating protein 6, was significantly lower in septic shock early after admission to the ICU compared to values measured in healthy subjects [33]. It can be assumed that bacterial co-infections in patients with COVID-19 also affect platelet aggregation; further research in this area is needed.

The observed decrease in platelet aggregation after stimulation with various agonists in patients with severe COVID-19 and ARDS may be multifaceted. Platelets play a role in the inflammatory response and are recruited to infection sites [34]. Autopsy data have shown that megakaryocytes in the bone marrow and lungs of COVID-19 patients were infected with the virus. More importantly, viral particles were detected in circulating platelets in blood [23]. This could result from either the active uptake of circulating viral proteins by platelets or the transfer from megakaryocytes. Koupenova et al. detected RNA from SARS-CoV-2 in platelets and demonstrated that platelets can rapidly internalize the virus either through ACE2 (angiotensin-converting enzyme) or by taking up virions attached to microparticles [35]. The presence of SARS-CoV-2 proteins within platelets was later confirmed by immunofluorescence and confocal microscopy [36]. The internalization process triggers platelet death programs, leading to the release of platelet contents and a reduction in their functionality. Another possible reason for the decreased platelet aggregation observed in our cohort is the early activation of platelets during inflammation, followed by partial desensitization. The overproduction of early response inflammatory cytokines, such as interleukin 6, interleukin 1β, and tumor necrosis factor, activates coagulation pathways, potentially leading to increased activation and consumption of platelet and coagulation factors. Several studies have reported platelet dysfunction in patients with COVID-19, including moderate thrombocytopenia and platelet hyperactivation [37,38]. Several studies indicate that platelets become activated and then partially desensitized during severe COVID-19. Garcia et al. observed platelet activation, as evidenced by elevated plasma levels of soluble platelet activation markers, including ligand for CD40, glycoprotein VI (GPVI), and P-selectin, in a cohort of patients with severe COVID-19 and ARDS [36]. The increased concentration of soluble GPVI in plasma was accompanied by a decreased amount of this receptor on the platelet surface due to proteolytic cleavage and receptor shedding during platelet activation. Additionally, there was a significant reduction in platelet glycoprotein IIb/IIIa activation and granule secretion in response to stimulation, suggesting the occurrence of a shedding process. Ex vivo testing of platelet reactivity revealed desensitization to physiological agonists. Another study confirmed partial desensitization during severe COVID-19, particularly in patients after a thrombotic event [39]. This observation was specific to platelet activation induced by the agonist thrombin receptor-activating protein 6 and may be explained by prior coagulation activation and exposure of platelets to high concentrations of thrombin. Consequently, the activation of PAR-1 (protease-activated receptor-1) may lead to platelet desensitization, rendering them less responsive to specific agonists. However, it is worth emphasizing that platelet aggregation via PAR-1 depends on the concentration of agonists used to activate the receptor, which may explain the differences in the results of aggregometry tests between studies [40].

Several studies have investigated platelet aggregation during ECMO therapy in the absence of SARS-CoV-2 infection, yielding conflicting results. Tauber et al. reported a 30% to 40% reduction in platelet function in response to stimulation with arachidonic acid, adenosine diphosphate, and thrombin receptor-activating protein 6 after 24 and 48 h on both VA- and VV-ECMO support [41]. Similarly, Nair et al. identified platelet abnormalities in VA- and VV-ECMO patients, with low aggregometry results, particularly after stimulation with ristocetin (61% of individuals) and ADP (72% of individuals) [42]. Kalbhenn et al. also observed the hypoaggregability of platelets after stimulation with ADP, ristocetin, collagen, and epinephrine in a cohort of patients on VV-ECMO support [43]. However, these studies did not exclude individuals with low platelet counts, and aggregometry analyses were often conducted on samples with low platelet counts, potentially affecting the results. A strong association between platelet count and platelet aggregation results using impedance aggregometry has been documented, leading to the test manufacturer’s recommendation that the platelet count in the tested samples should exceed 100,000 [44]. Balle et al. later found that platelet aggregation during ECMO support was primarily impaired due to low platelet counts, with patients having platelet counts below 50 × 10^9^/L showing the lowest levels of aggregation following stimulation with arachidonic acid, ADP, and thrombin receptor-activating protein 6 [45]. Consequently, Balle’s findings suggest that platelet aggregation is not significantly impaired during ECMO when considering platelet count in the tested sample.

Patients undergoing ECMO treatment have a standardized method of anticoagulant therapy, which includes a continuous infusion of heparin, either UFH or LMWH. The risk of complications during ECMO is high and is related both to factors associated with the patient’s clinical status and the treatment itself. Finding safe and effective anticoagulants that balance thrombosis and bleeding risk remains challenging during ECMO support [46]. Modification of anticoagulant therapy during ECMO with next-generation anticoagulants might be beneficial; however, clinical research and trials are required to determine the optimal anticoagulant dosage in both adult and pediatric patients [47]. Early diagnosis of coagulation complications is crucial during ECMO, and several monitoring methods, including POCT (point of care testing) and laboratory-based equipment, have been developed to track the patient’s coagulation status and adjust the anticoagulant dosage. Whole blood impedance aggregometry is one of the reliable and quick POCT methods that can be used to measure platelet aggregation in response to different agonists to support other conventional coagulation monitoring methods in ECMO.

This study has several limitations. It is a single-center analysis; the results are based on data from a single center, which limits the generalizability of the findings. Given that the COVID-19 epidemic has ended, the results cannot be verified in larger cohorts. Another limitation of this study is that while the results demonstrate a decrease in platelet aggregation in patients undergoing V-V ECMO, they do not offer a mechanistic explanation. Specifically, it remains unclear whether the reduced platelet responsiveness is attributable to platelet infection by SARS-CoV-2 or a loss of platelet glycoprotein, which was not quantified in this study. Additionally, all analyses were based on data from hospital records, and platelet activity tests were not part of the standard treatment for COVID-19 patients outside the ICU. Therefore, the explanation suggesting that reduced platelet aggregation is a consequence of hyperactivation in the period before ICU admission is hypothetical, and based on the available literature, it needs to be confirmed [36,39]. Future studies should replicate these analyses in ICU patient cohorts with viral infections other than COVID-19 to validate the findings.

## 5. Conclusions

In summary, most patients exhibited reduced platelet aggregation upon ICU admission, which further decreased over a 5-day observation period, likely due to prior coagulation activation followed by partial platelet desensitization. Our data suggest that platelet hypo-responsiveness was a common feature that aggravated disease progression and was associated with increased mortality.

## Figures and Tables

**Table 1:** Patient baseline characteristics.

Parameter	All	Survivors	Nonsurvivors	*p*
	N = 28	N = 13	N = 15	
**Age**	44 (35–55)	37 (33–45)	53 (39–57)	0.664
**Gender, female/male (N)**	8/25	5/8	3/12	0.255
**Coexisting conditions, n (%)**				
Coronary heart disease	2 (7)	0	2 (13)	0.277
Hypertension	3 (28)	1 (8)	2 (13)	0.555
Diabetes mellitus	2 (7)	0	2 (13)	0.277
Stroke	1 (4)	1 (8)	0	0.464
Asthma	2 (7)	0	2 (13)	0.277
Obesity	6 (25)	3 (23)	4 (27)	0.587
**APACHEII score**	14 (10–18)	13 (10–15)	16 (12–21)	0.169
**SOFA score**	9 (8–11)	9 (8–10)	10 (7–12)	0.427
**Interleukin 6 (pg/mL)**	47.0	20.5	110.7	0.041
	(19.6–214.0)	(12.5–84.7)	(45.3–447.3)	
**C-reactive protein (mg/dL)**	142.0	146.0	141.0	0.645
	(81.0–217.4)	(67.0–216.0)	(83.0–266.0)	
**Septic shock**	21 (75)	11 (85)	10 (67)	0.274
**RRT**	4 (14)	0	4 (27)	0.044
**ICU LOS (day)**	23 (16–40)	30 (24–43)	18 (12–39)	0.033
**Hospital LOS (day)**	26 (19–48)	35 (25–59)	19 (12–45)	0.050

LOS, length of stay. Variables are presented as medians with the 25th and 75th percentiles. *p*-value represents differences between survivors and nonsurvivors.

**Table 2:** Percentage of patients with platelet aggregation below the reference range after stimulation with arachidonic acid (ASPI), adenosine diphosphate (ADP), thrombin receptor-activating protein 6 (TRAP), and ristocetin (RISTO) measured in patients on days 1, 3, and 5 of ECMO treatment.

	ASPI < 745 AU/min	ADP < 534 AU/min	TRAP < 941 AU/min	RISTO < 896 AU/min
	N (%)	N (%)	N (%)	N (%)
**1**	19 (68)	17 (61)	25 (89)	19 (68)
**3**	23 (82)	21 (75)	26 (93)	19 (68)
**5**	23 (82)	21 (75)	25 (89)	22 (79)

**Table 3:** Coagulation parameters by group on days 1, 3, and 5.

	Day	Survivors	Nonsurvivors	*p*
**Platelet count (×10^9^/L)**	1	267 (228–309)	261 (190–358)	0.747
ref. range 145–400 × 10^9^/L	3	231 (179–272)	190 (147–258)	0.197
	5	256 (167–267)	177 (105–221)	0.105
**D-dimer (mg/L)**	1	5.3 (1.9–7.0)	3.9 (1.8–17.5)	0.998
ref. range 0–0.5 mg/L	3	2.3 (1.7–4.7)	6.5 (3.1–11.5)	0.021
	5	4.5 (2.3–6.8)	10.8 (2.0–15.9)	0.024
**Fibrinogen (g/L)**	1	5.5 (4.2–7.8)	6.1 (3.2–7.8)	0.871
ref. range 1.8–3.5 g/L	3	4.4 (4.3–6.7)	5.8 (3.7–7.1)	0.644
	5	5.9 (4.5–6.7)	5.9 (4.1–6.9)	0.844
**AT III (%)**	1	93 (73–109)	85 (78–98)	0.835
80–120%	3	80 (70–91)	80 (69–93)	0.980
	5	80 (71–104)	88 (66–95)	0.644

AT III, antithrombin III. Variables are presented as medians with the 25th and 75th percentiles. *p*-value represents differences between survivors and nonsurvivors.

**Table 4:** Thromboelastometry parameters by group on days 1, 3, and 5.

Parameter	Day	Survivors	Nonsurvivors	*p*
**EXTEM**				
CT (sec)	1	69 (63–75)	66 (58–91)	0.857
ref. range 38–79 s.	3	71 (60–74)	76 (64–90)	0.336
	5	59 (54–81)	76 (64–81)	0.219
CFT (sec)	1	58 (55–63)	63 (55–77)	0.180
ref. range 34–159 s.	3	50 (49–61)	69 (52–75)	0.081
	5	61 (47–66)	93 (61–134)	0.066
MCF (mm)	1	69 (68–72)	71 (67–74)	0.959
ref. range (50–72 mm)	3	71 (68–72)	68 (65–72)	0.334
	5	69 (62–73)	65 (55–71)	0.347
**INTEM**				
CT (sec)	1	246 (206–373)	242 (160–293)	0.368
ref. range 100–240 s.	3	228 (203–381)	209 (186–284)	0.382
	5	198 (197–215)	199 (152–251)	0.683
CFT (sec)	1	66 (60–82)	63 (56–79)	0.571
ref. range 30–110 s.	3	67 (59–93)	71 (60–83)	0.789
	5	63 (59–71)	89 (64–123)	0.153
MCF (mm)	1	68 (66–72)	70 (66–73)	0.536
ref. range (70–83 mm)	3	69 (68–72)	66 (64–73)	0.594
	5	68 (62–72)	65 (54–71)	0.268

CT, clotting time; CFT, clot formation time; MCF, maximum clot firmness. Variables are presented as medians with the 25th and 75th percentiles. *p*-value represents differences between survivors and nonsurvivors.

## Data Availability

The data presented in this study are available upon request from the corresponding author. The data have not been made publicly available because they contain information that could compromise the privacy of the study participants.
